# Temperature data during steam pressure filtration in combination with a water insoluble pore liquid

**DOI:** 10.1016/j.dib.2020.105812

**Published:** 2020-06-03

**Authors:** Simon Esser, Urs A. Peuker

**Affiliations:** Institute of Mechanical Process Engineering and Mineral Processing, TU Bergakademie Freiberg, Agricolastraße 1 – 09599 Freiberg - Germany

**Keywords:** filter cake dewatering, steam pressure, volatile organic compound, steam stripping, steam distillation, thermal drying, temperature profile

## Abstract

Data presented in this article focus on the application of steam pressure filtration in combination with a water insoluble pore liquid. The article describes measured temperature profiles during steam pressure filtration within a filter cake. This article is co-submitted to the article ‘Steam Pressure Filtration in Combination with a Water Insoluble Pore Liquid’ [1] (DOI: 10.1016/j.ces.2020.115782) where the occurring phenomena as well as the interpretation of temperature profiles during steam pressure filtration are explained in detail. The article expands the shown data to other material systems.

Specifications Table

**Table:** 

Subject	Filtration and Separation
Specific subject area	Purification of a filter cake loaded with a volatile organic compound using steam pressure filtration
Type of data	Tables Figures
How data were acquired	Filter cakes are treated with steam in a nutsche filter for steam pressure filtration. Thermocouples measure the temperature and data is recorded in LabView (National Instrument)
Data format	Raw Data, analysed
Parameters for data collection	Suspension of VOC and solid (volume concentration 0.15) Cake formation with pressurized nitrogen (pressure difference according to steam pressure, cake height 20 mm) Displacement of pore liquid with steam until steam breakthrough (different pressure differences) Steam stripping by further steam flow Steam drying by further steam flow
Description of data collection	Thermocouples (Ni-Cr Ni) within the filter cake in different layers measure the temperature during the whole process. The temperature data is recorded with LabView (National Instruments)
Data source location	Institute of Mechanical Process Engineering and Mineral Processing, TU Bergakademie Freiberg Freiberg Germany
Data accessibility	With the article
Related research article	Simon Esser, Urs A. Peuker, Steam pressure filtration in combination with a water insoluble pore liquid, Chemical Engineering Science, In Press

Value of the Data

• The temperature data are useful to validate the applicability of steam stripping (steam distillation) during steam pressure filtration.
• The process provides an alternative to conventional dewatering and drying processes to treat solids contaminated with volatile organic compounds and thus operators of the completely chemical process industry (i.e. purification of solids after crude oil extraction, polymerization processes or multistep syntheses) as well as filter manufacturers could benefit from these data.
• The data can be used for process understanding and the experimental design to verify the feasibility of steam pressure filtration for the desired material system.
• The data potentially helps drawing attention on steam pressure filtration, which possibly provides an energy saving alternative to conventional process combinations of mechanical separation and thermal drying processes.

## 1 Data Description

**Fig. 1:**
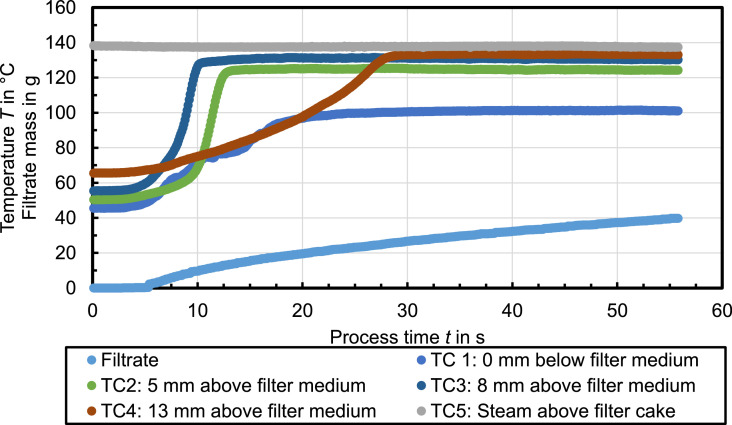
Celite®512 & Water (Δ*p* = 2.4 bar, *T*_st_ = 138°C, *h*_FC_ = 20 mm); *RM* = 48.5 %, *r*_C_ = 4 × 10^13^ 1/m²

Presented data in this article deal with the characteristic temperature profiles during steam pressure filtration [1], [2], [3]. The temperature data in Fig. 1 to Fig. 7 illustrate exemplary temperature profiles within a filter cake during steam pressure filtration. Fig. 1 shows the temperature measurement for water as mother liquid. The temperature rapidly increase stepwise in all filter cake layers to a certain steady state temperature plateau. The temperature at thermocouple TC1 immediately below the filter medium increases to *T*_st_ = 100°C which is the boiling temperature of steam at ambient pressure. The residual moisture of the filter cake after 37 s steam treatment is *RM* = 48.5 %.

**Fig. 2:**
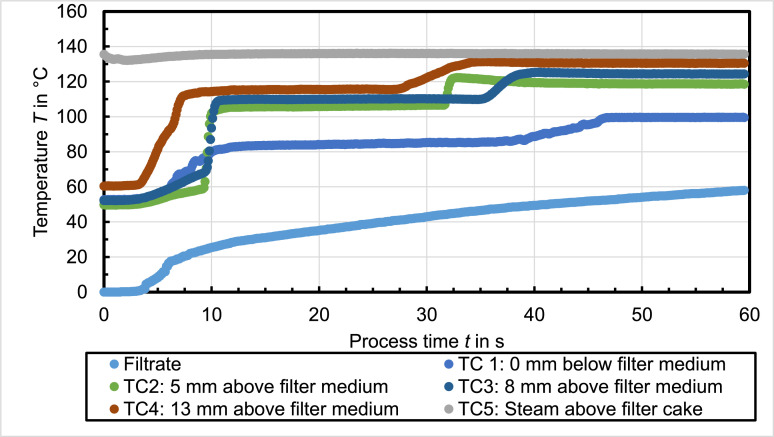
Celite®512 & toluene (Δ*p* = 2.4 bar, *T*_st_ = 138°C, *h*_FC_ = 20 mm); *RM* = 16 %, *r*_C_ = 9 × 10^12^ 1/m²

Fig. 2 shows the temperature profile occurring when the mother liquid is insoluble in water, which is toluene in this case. The temperature in all layers of the filter cake also increase rapidly, but before they reach the steady state, they remain on a second temperature plateau for a certain time, which is lower compared to the steady state temperature. The temperature plateaus measured at TC1 at ambient pressure are *T*_vap,1_ ≈ 84°C and *T*_vap,2_ = 100°C.

The filtrate mass increases linearly until the temperature at TC1 begins to rise. Afterwards, the filtrate flow flattens before the increase is again linear when the temperature at TC1 reaches the steady state. The residual moisture of the filter cake after 40 s steam treatment amounts *RM* = 16 %.

**Fig. 3:**
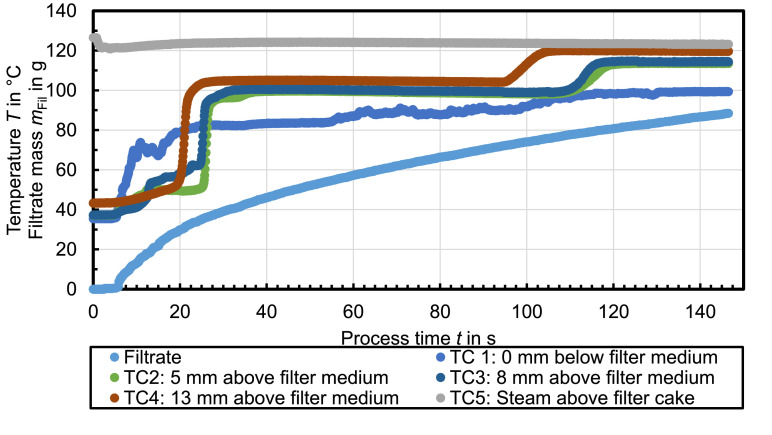
Celite®512 & toluene (Δ*p* = 1.3 bar, *T*_st_ = 120°C, *h*_FC_ = 20 mm); *RM* = 20 %, *r*_C_ = 3 × 10^12^ 1/m²

Fig. 3 shows a similar temperature profile compared to Fig. 2 but the steam pressure above the filter cake is set to T_st_(TC5) = 120°C. Nevertheless, the temperature plateaus measured at TC1 at ambient pressure are *T*_vap,1_ ≈ 84°C and *T*_vap,2_ = 100°C. The filtrate mass increase is similar compared to the shown profile before but more filtrate is collected although the residual moisture is higher (*RM* = 20 %). Furthermore, the process time, or rather the duration until the steady state is reached, is longer for a lower applied pressure difference or steam temperature, respectively.

**Fig. 4:**
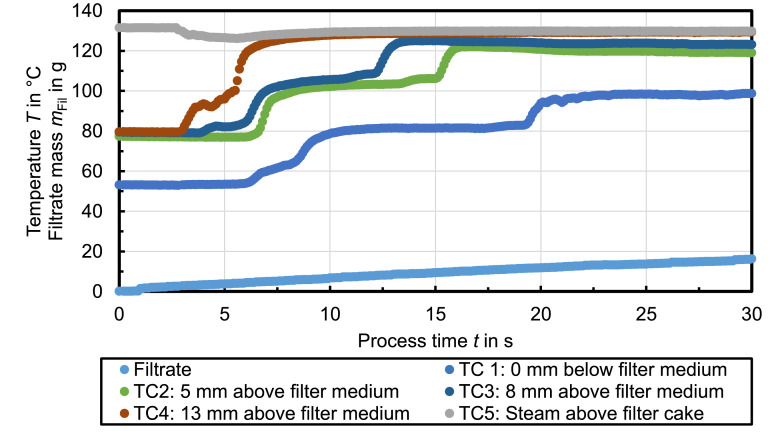
Celite®512 & n-heptane (Δ*p* = 1.7 bar, *T*_st_ = 130°C, *h*_FC_ = 20 mm); *RM* = 7.4 %, *r*_C_ = 9 × 10^12^ 1/m²

Fig. 4 shows the recorded temperature profiles during steam pressure filtration with n-heptane used as mother liquid. The temperature plateaus measured at TC1 at ambient pressure are *T*_vap,1_ ≈ 82°C and *T*_vap,2_ = 100°C. The first temperature plateau is not constant but slightly increasing. The filtrate mass profile is similar than before but the total filtrate mass is rather low, although the residual moisture of the filter cake after 30 s steam treatment is *RM* = 7.4 %.

**Fig. 5:**
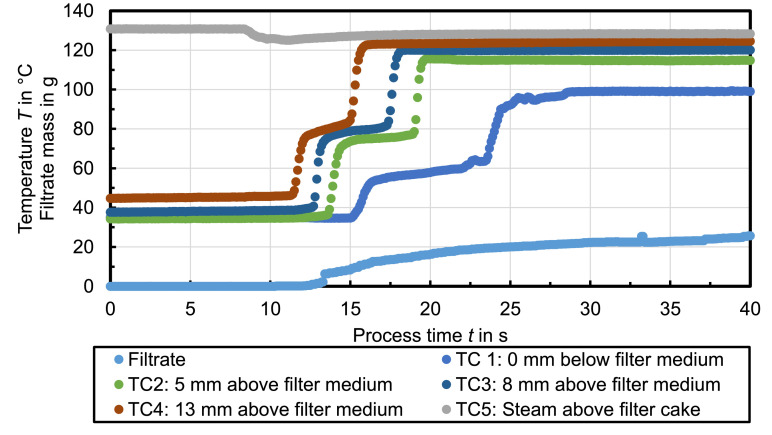
Celite®512 & n-hexane (Δ*p* = 1.7 bar, *T*_st_ = 130°C, *h*_FC_ = 20 mm); *RM* = 22 %, *r*_C_ = 1 × 10^13^ 1/m²

Fig. 5 shows the recorded temperature profiles during steam pressure filtration with n-hexane used as mother liquid. The temperature plateaus measured at TC1 at ambient pressure are *T*_vap,1_ ≈ 60°C and *T*_vap,2_ = 100°C. The filtrate mass profile is similar to the upper described experiments. The steam treatment of the filter cake for 40 s leads to a residual moisture of *RM* = 22 %.

**Fig. 6:**
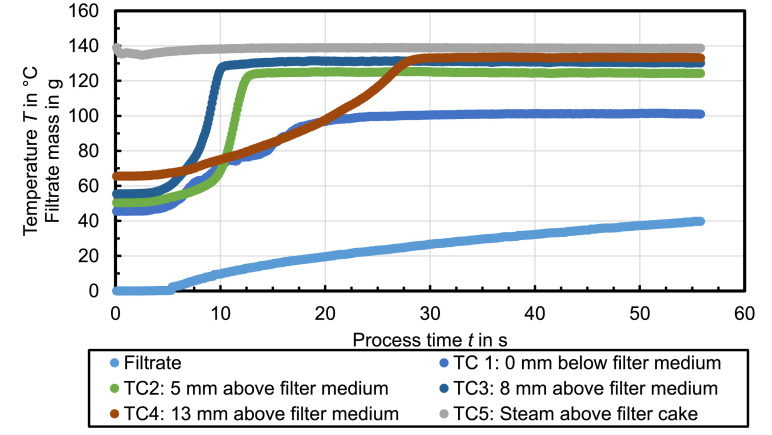
Celite®512 & n-dodecane (Δ*p* = 2.7 bar, *T*_st_ = 140°C, *h*_FC_ = 20 mm); *RM* = 35.1 %, *r*_C_ = 1 × 10^12^ 1/m²

Fig. 6 shows the recorded temperature profiles during steam pressure filtration with n-dodecane used as mother liquid. In this case only one temperature plateau is measured at TC1 at ambient pressure (*T*_vap,1_ = 100°C). The residual moisture after 50 s of steam treatment is *RM* = 35.1 %.

**Fig. 7:**
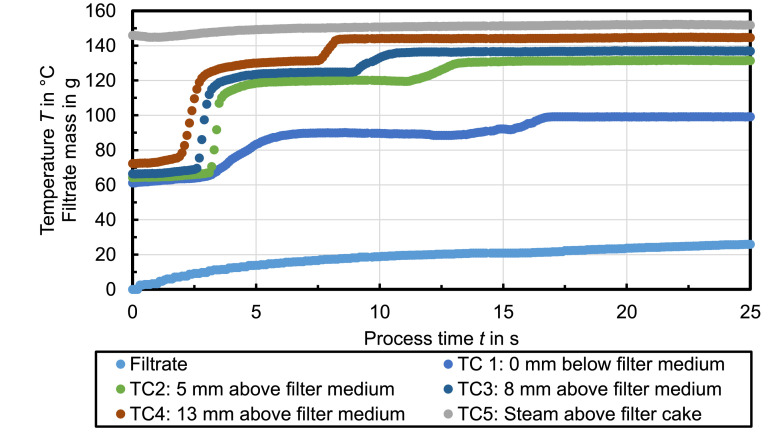
Celite®512 & n-octane (Δ*p* = 3.7 bar, *T*_st_ = 150°C, *h*_FC_ = 20 mm); *RM* = 26.5 %, *r*_C_ = 2 × 10^12^ 1/m²

Fig. 7 shows the recorded temperature profiles during steam pressure filtration with n-octane used as mother liquid. The temperature plateaus detected at TC1 at ambient pressure are *T*_vap,1_ ≈ 89°C and *T*_vap,2_ = 100°C. The steam treatment of the filter cake for 25 s results in a residual moisture of RM = 26.5 %.

## 2 Experimental Design, Materials, and Methods

The following chapter is an extended version of the chapter Materials & Methods of the main publication ‘Steam Pressure Filtration in Combination with a Water Insoluble Pore Liquid’ [1].

**Fig. 8:**
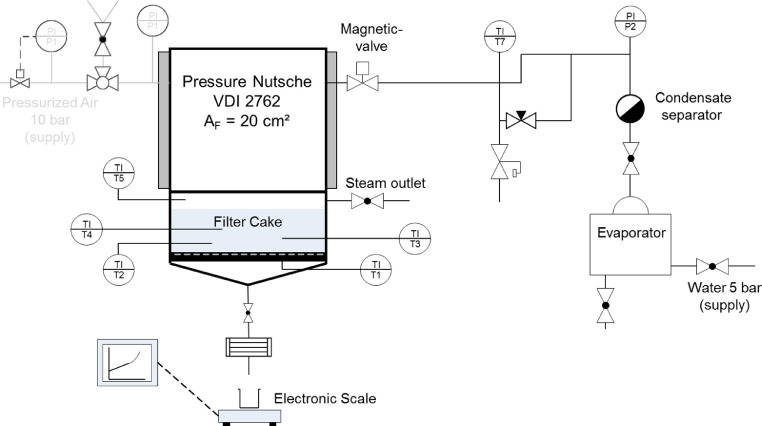
Flow diagram of steam pressure filtration equipment with pressure nutsche according to VDI Guideline 2762-2 [4]

The experiments are carried out in a modified pressure nutsche in accordance with VDI Guideline 2762 [4]. The flow diagram of the steam pressure filtration plant is shown in Fig. 8. The core of the system is the temperature controlled, double-walled, cylindrical nutsche filter.

The right part in the flow diagram shows the preparation of steam. To provide saturated steam, water is evaporated and the entrained liquid water from the evaporator is separated in the condensate separator, which is placed in the standpipe. Thereby the steam is passed through a bed of steel wool. The drops are separated due to their inertia and collected in a container. The steam emerges from the separator in a saturated state. The pipes are heated to avoid a further condensation of the steam at the pipe walls. The steam can also be slightly superheated if the temperature of the pipe heating is higher than the boiling temperature of the steam.

**Fig. 9:**
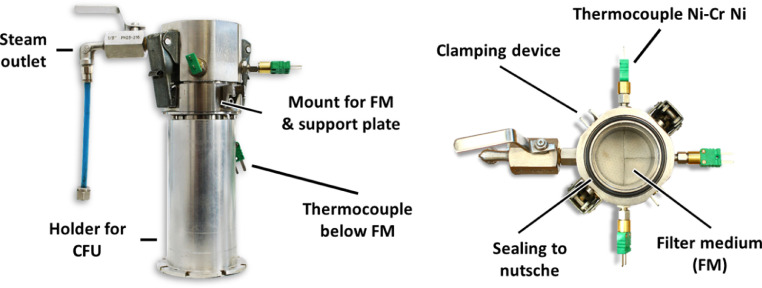
Cake formation unit – left: side view of the cake formation unit on a holder, right: top view

The lower part of the nutsche consists of a removable cake formation unit, which can be clamped with quick-locks to the nutsche. The cake formation unit is also shown in Fig. 9.

It is composed of a drain funnel and the so-called cake forming ring. The funnel also serves as a mount for support plate and filter medium, which is clamped between mount and cake-forming ring with quick-release fasteners. The thermocouple TC1 is in contact with the filter medium and thus measures the temperature of the filtrate at the outlet at ambient pressure. Additionally, thermocouples TC2-TC5 are implemented to measure the temperature at different layers in the filter cake and above. The cake-forming ring is also equipped with a valve for discharging air when steam is applied. The top cover of the nutsche has a sight glass. Using a light it is possible to observe the suspension and filter cake during the process.

The experiments are carried out in the described steam pressure filtration equipment. The nutsche is first flushed with nitrogen before the suspension is filled into the heated nutsche. The solids volume concentration for all experiments is *c*_V_ = 0.15The top cover is closed and pressurized nitrogen is applied. Nitrogen is used due to safety reasons to avoid explosive gas mixtures that might occur during the filter cake formation. The suspension is forced in direction of the filter medium (JunkerFilter JF 7945). The filter cake formation is finished as soon as the liquid surface reaches its surface (*S* = 1) and the nutsche is vented.

The filter cake formation is evaluated according to VDI Guideline 2762 [4], which is essentially based on the linear evaluation of the *DARCY*-equation (1) [5]. Therefore, the *t*/*V* – *V* plot is used to calculate the specific filter cake resistance and the filter medium resistance assuming that the filter cake is incompressible.

(1) $$\frac{t}{V_{Fil}}=\frac{\eta \;r_{c\;}\kappa }{2\;A_{F}\;\Delta p}V_{Fil}+\frac{\eta \;R_{M}}{A_{F}\;\Delta p}$$

In the next step, the steam outlet and the magnetic valve are opened to allow the steam to enter the process room. When steam flows out of the steam outlet it is closed (*t* < 2 s). The steam begins to penetrate the filter cake. The advance of the condensation front is displayed by the temperature profiles measured with the thermocouples. The liquid/ gas mixture from the filter flows through a condenser to ensure a liquid filtrate, which is collected in a beaker and measured by a scale.

The steam filtration experiments are ended after a defined process time by closing the magnetic valve of the steam inlet. The nutsche is carefully vented to avoid the expansion of the condensate within the pores of the filter cake, which could lead to the destruction of the filter cake. The cake formation unit is removed after the experiment from the nutsche and small axial samples are taken with a sampling spear (*d* = 5 mm). The samples are dispersed in isopropyl alcohol (IPA). The composition of the vapour phase above the liquid mixture (VOC/ water/ IPA) is analysed by headspace gas chromatography (GC). Using a suitable calibration and combining these results with a gravimetric analysis, the composition of the liquid mixture and thus the composition of the filter cake can be determined.

**Fig. 10:**
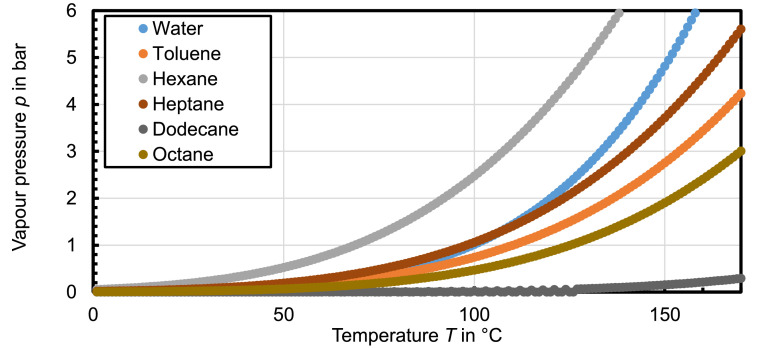
Vapour pressure curves of all used fluids [8, 10, 11, 15]

The solids used in this work are diatomaceous earth (Celite®512) representing a naturally occurring material with inner pores leading to high residual moistures after conventional dewatering. The solids are sylanized (Dynasylan® OCTEO) improving wettability for the solvent [6]. The liquids used as pore liquid are toluene, n-hexane, n-heptane, n-dodecane and n-octane (Carl Roth, 99%), which are poorly miscible with water. Further information of the used liquids are given in [7, 8] for water, in [9, 10] for toluene, in [11], [12], [13] for n-hexane, in [10, 14] for n-dodecane and in [11, 13] for n-heptane and n-octane. In addition, the vapour pressure curves for all used fluids are displayed in Fig. 10

Symbol directory

**Table:** 

**Latin symbols**			**Indices**	
*S*	[-]	Degree of saturation	FC	Filter cake
*h*	[m]	Height	st	Steam
Δ*p*	[Pa]	Pressure difference	c	Cake
*T*	[K]	Temperature	F	Filter
*r*	[1/m²]	Specific resistance	M	Filter medium
*c*	[-]	Solids volume concentration	V	Volume
*p*	[Pa]	Absolute pressure	vap	Vapour mixture
*RM*	[%]	Residual moisture	Fil	Filtrate
*m*	[kg]	Mass		
*V*	[m³]	Volume		
*A*	[m²]	Area		
*t*	[s]	Time		
*d*	[m]	Diameter		
*R*	[1/m]	Resistance		
**Greek symbols**				
*η*	[Pa s]	Dynamic viscosity		
*κ*	[-]	Concentration coefficient		

## Declaration of Competing Interest

The authors declare that they have no known competing financial interests or personal relationships, which have, or could be perceived to have, influenced the work reported in this article.
